# Evaluation of intracellular anion superoxide level, heat shock protein A2 and protamine positive spermatozoa percentages in teratoasthenozoospermia

**Published:** 2017-05

**Authors:** Parvin Sabeti, Fardin Amidi, Seyed Mahdi Kalantar, Mohammad Ali Sedighi Gilani, Soheila Pourmasumi, Atefeh Najafi, Ali Reza Talebi

**Affiliations:** 1 *Department of Anatomy, School of Medicine, Kurdistan University of Medical Sciences, Sanandaj, Iran.*; 2 *Department Of Anatomy, School of Medicine, Tehran University of Medical Sciences, Tehran, Iran.*; 3 *Research and Clinical Center for Infertility, Yazd Reproductive Sciences Institute, Shahid Sadoughi University of Medical Sciences, Yazd, Iran. *; 4 *Department of Urology, School of Medicine, Tehran University of Medical Sciences, Tehran, Iran.*

**Keywords:** Male infertility, Sperm chromatin, HSPA2, Protamine deficiency

## Abstract

**Background:**

Teratoasthenozoospermia (TA) is a severe form of male infertility with no clear etiology.

**Objective:**

To compare the level of intracellular anion superoxide (O_2_–), heat shock protein A2 (HSPA2) and protamine deficiencies in ejaculated spermatozoa between teratoasthenozoospermic and normozoospermic men.

**Materials and Methods:**

In this case- control study, semen samples of 20 infertile men, with TA (with normal morphology lower than 4%_ and total motility lower than 40% ) as the case group and 20 normozoospermic fertile men as the control group were evaluated for intracellular O_2_^–^ and HSPA2 by flow cytometry and protamine deficiency by Chromomycin A3 (CMA3) test.

**Results:**

The rate of CMA3+ spermatozoa in the case group was higher than controls (p=0.001). The percentages of HSPA2+ spermatozoa in the cases were significantly lower than controls (p=0.001). Also, intracellular O_2_^–^ levels in the case group were significantly higher than controls (p=0.001) and had positive correlations with sperm apoptosis (r=0.79, p=0.01) and CMA3 positive sperm (r=0.76, p=0.01), but negative correlations with normal morphology (r=-0.81, p=0.01) and motility (r=-0.81, p=0.01). There was no significant correlation between intracellular O_2_^–^ and HSPA2 in the case group (r=0.041, p=0.79).

**Conclusion:**

We suggest that the increase in intracellular O_2_^–^, decrease in spermatozoa HSPA2^+^, and high percentages of spermatozoa with immature chromatin might be considered as etiologies of infertility in TA patients.

## Introduction

Mammalian spermatozoa have the capability to produce various reactive oxygen species (ROS), which are involved in the physiological functions, such as sperm capacitation, oocyte fusion and acrosomal reaction (1). However, these short-lived and highly reactive radicals could potentially compromise lipid and protein content of human spermatozoa, sperm DNA and also sperm function (2). Overproduction of ROS and imbalance between ROS production and antioxidants in semen are the causing factors of oxidative stress, which in turn deteriorate spermatozoa function (4). In addition, there is a relationship between ROS generation and spermatozoa immaturity: there is also a relation between ROS-induced oxidative stress and male infertility (4-6). Infertility has an incidence of about 8% among reproductive age males, and there is a higher production rate of ROS by abnormal spermatozoa with poor motility and morphology in infertile patients than in fertile males with normal functional cells (3, 7, 8).

Anion superoxide (O_2_^−^) has been identified as the primary free radical of spermatozoa (2, 9). The O_2_^−^ is produced by spermatozoa in a commonly rapid and temporary rate, and it is the main stimulator of lipid peroxidation in spermatozoa plasma membrane (2, 9). Mazzilli *et al* demonstrated that infertile males have a high level of O_2_^−^, and this radical exerts the toxic effect on spermatozoa. The severity of this effect depends on the exposure time period and concentration of O_2_^−^ (10).

Heat shock protein A2 (HSPA2) is a molecular chaperone that assembles in mitochondria, cytoplasm, and reticulum endoplasmic. This molecule has been recognized to have a crucial role in male reproduction, including sperm-egg recognition, protein packaging, and transportation, substitution of histones by protamines during spermiogenesis, inhibition of apoptosis and removal of remained cytoplasm during sperm maturation (11-15). Several recent studies have shown the possible association between HSPA2 and male factor infertility (11, 16).

In fact, reduced expression of HSPA2 is associated with cytoplasmic retention and the resulting excess residual cytoplasm (ERC), has a positive correlation with ROS generation (15, 17). As a result, high levels of ROS, cause to a condition of oxidative stress which characterized by damage to mitochondrial and nuclear DNA (17, 18). HSPA2 has a probable ability to predict in vitro fertilization pregnancy outcomes (19). Also, it has been shown that the depression of HSPA2, is associated with spermatogenic and fertilization impairment in intracytoplasmic injection for azoospermic patients (20). 

We suggest that the alternations in the level of intracellular anion superoxide, percentages of HSPA2 and protamine positive spermatozoa might be causes of infertility in terato-asthenozoospermic men. So, we aimed to compare these factors in ejaculated spermatozoa between normozoospermic and idiopathic TA patients. 

## Materials and methods

In this case-control study, semen samples were provided from 40 men referred to andrology laboratory of Royan Research and Clinical Center for Infertility (Tehran, Iran) from June 2014 to June 2016. 

Semen samples were evaluated in two groups. The case group consisted of 20 infertile men with terato-asthenozoospremia (with normal morphology lower than 4% and total motility lower than 40%). A complete medical history was collected from each participant. Cases with leukocytospermia (>1× 10^6^ WBC/ml), cancer, varicocele, genitourinary inflammation, endocrine disorders, autoimmune disease, cryptorchidism and subjects who were smoking or had alcohol consumption which may impact the intracellular O_2_^−^ were excluded from this study. Also, at the time study began, none of the cases had been pre- treated with antioxidants. The control group consisted of fertile men comprised healthy, normozoospermic (with the same exclusion criteria) whose their partners had successful pregnancies within the last one year. 

**Sperm collection and analysis**

Semen samples were obtained by masturbation after sexual abstinence of 2-4 days. After liquefaction, they evaluated for sperm parameters according to World Health Organization (WHO) criteria (21). 

**Assessment of sperm viability and morphology**

Sperm viability and morphology in 200 spermatozoa per slide were assessed by Eosin/Nigrosin test and Papanicolaou staining, respectively. 

**Flow cytometry analysis for evaluation of intracellular** **O**_2_^−^ **, HSPA2, and apoptotic spermatozoa**

The o_2_^−^ assessment was performed using DHE (Dihydroethidium) (22). Sperm suspension was incubated with 1.25 µM DHE (DHE; Sigma) at 25ºC for 20 min. The intracellular O_2_^−^ oxidizes DHE and produces ethidium bromide which binds to the DNA and emits red fluorescence, then it analyzed via flow-cytometry (FACS Calibur; BD Biosciences, USA) between 590 and 700 nm. apoptotic spermatozoa were excluded by Yo- pro -1 Iodide (Y3603- Life Technology ) as a counterstaindye for the DHE (22). In this study at least 10000 spermatozoa were assessed for each sample, and data from flow- cytometry were interpreted using Flow-cytometry software (Flowjo 7.6.1) and expressed in percentage.

To analyze HSPA2, samples were washed twice with phosphate-buffered saline (PBS, Gibco, USA), fixed with 4% paraformaldehyde for 20 min at room temperature, and centrifuged for 5 min at 300g. After permeability in 0.5% Triton X-100 for 5 min, test fractions were incubated overnight with the primary anti-HSPA2 antibody (Santa Cruz Co.) at a dilution of 1:100 in 3% bovine serum albumin (BSA; Sigma Co.) at 4^o^C. Control samples were incubated under the same conditions with 3% BSA. Two samples were washed and incubated with PE- conjugated Donkey anti-goat IgG (1:200, Santa Cruz Co.) in 1.5% BSA at 4^o^C for 1 hr. After washing, BD FACS Calibur flow-cytometry was used for further analysis (16). 

**Chromomycin A3 (CMA3) staining for Protamine deficiency assessment**

**Figure 1:**
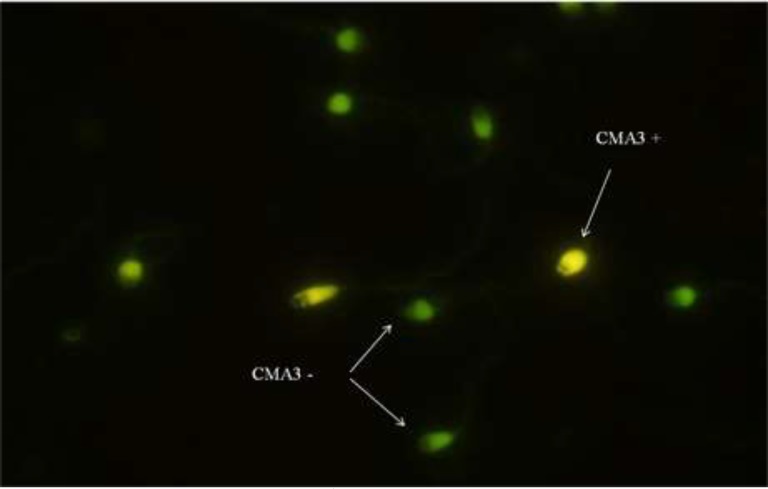
CMA3 staining: Bright yellow sperm cells (CMA3^+^) show protamine deficiency and yellowish green sperm cells (CMA3^-^) show normal protamine content (fluorescent microscopy, ×100 eyepiece magnification).

Chromomycin A3 ( CMA3), is an indirect assessment of protamine deficiencies, which competes with protamine to bind DNA (23). It is used for the estimate of the percentage of protamination in sperm chromatin. To do this test, semen samples were washed with PBS, and smears of samples were prepared and dried. Following fixation in Carnoy’s solution (Methanol/Glacial acetic: 3:1) at 4^o^C for 10 min, slides were stained with 100 µl of CMA3 (0.25 mg/ml) (Sigma Co.) for 25 min in dark at room temperature and mounted with buffered glycerol. Then, 200 spermatozoa were counted under a fluorescence microscope (BX51, Olympus, Tokyo, Japan) at 1000× magnification with oil immersion. Protamine deficient spermatozoa (CMA3-positive) and normal protamine spermatozoa (CMA3-negative) are staining bright yellow and a dull green (Figure1), respectively.

**Ethical consideration**

This study was approved by institutional review board of Yazd Research and Clinical Center for Infertility and informs consent forms signed by all participants.

**Statistical analysis**

The data were presented as mean±SEM, and significant level was defined at p<0.05. Kolmogorov-Smirnov test (K-S, #1862) was used for evaluating normal distribution for quantitative data. Independent-samples t-test was applied in order to compare the parameters between experimental groups. Pearson test was performed for evaluation of the correlation between the percentage of O_2_^−^ positive sperm and other variables. Statistical analysis was carried out using Stastical Package for the Social Sciences, version 16.0, SPSS Inc, Chicago, Illinois, USA ( SPSS software

## Results

There was no significant difference between mean age in case and control groups (33.7±6.8 vs 32.1±5.7, respectively, p=0.173). Sperm concentration, total motility, morphology, vitality and total sperm count were analyzed according to WHO guidelines and reported in table I. 

**Flow cytometry analysis of** **O**_2_^−^ **and apoptotic spermatozoa**

**Figure 2:**
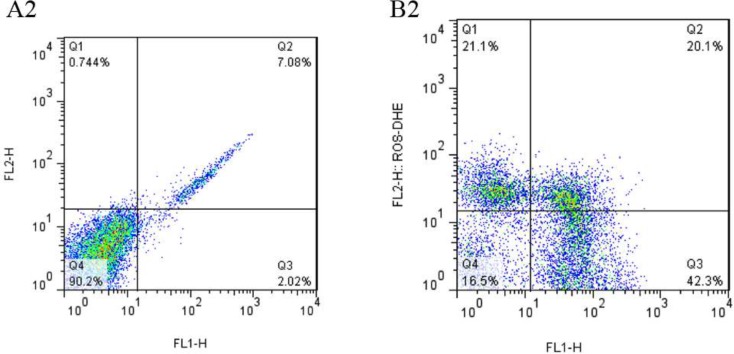
Dot plot flow- cytometry histograms, showing the intracellular O_2_^−^ levels measured by DHE in normozoospermic (control) (A2), and terato- asthenozoospermic (case) men (B2). The lower left quadrant represents viable, non-stained sperm and lower right represents apoptotic sperm. The upper left quadrant represents viable sperm with high intracellular O_2_^−^, and upper right shows apoptotic sperm with high intracellular O_2_

A minimum number of 10000 spermatozoa per sample were analyzed by flow cytometer FACS can (Becton Dickinson). Flow-cytometry data were analyzed with Flowcytometer Software (FlowJo 7.6.1). The percentage of apoptotic spermatozoa (YO+) and level of intracellular O_2_^−^ were significantly higher in the case group than controls (Table II). Dot plot flow-cytometry histograms by flow-cytometry in two groups are shown in figure 2. 

**Flow cytometry analysis of HSPA2, protamine deficiency and semen parameters**

The percentage of spermatozoa HSPA2^+^ was significantly lower in the case group than controls, while intracellular O_2_^−^ was higher in case group (Table II). Also, the percentage of spermatozoa with protamine deficiencies was higher in the case group than controls (Table II). Intracellular O_2_^−^ revealed positive correlations with CMA3_positive sperm (r=0.76, p=0.01) and sperm apoptosis (r=0.79, p=0.01) but, negative significant correlations with sperm normal morphology (r=-0.81, p=0.01), viability (r=-0.83, p=0.01), motility (r=-0.81, p=0.01), sperm count (r=-0.83, p=0.01) and sperm concentration (r=-0.052, p=0.05).

**Table I:** The result of sperm parameters in terato- asthenozoospermic (case) and normozoospermic (control) men (n=20/each

**Variables**	**Control group **	**Case group **	p-value*
Concentration (×10^6^ ml)	87.62±7.09	43.75±7.51	0.001
Total motility (%)	72.10±3.13	28.73±1.49	0.001
Normal morphology (%)	7.12±0.8	2.2±0.31	0.001
Vitality (%)	86.38±2.86	55.63±5.41	0.001
Total sperm count (×10^6^)	121.54±30.38	97.32±24.33	0.01

Values are mean±SE.

* independent-samples t-test

**Table II:** The results of the level of interacellular O_2_^-^ (DHE^+^), percentage of spermatozoa CMA3^+^, HSPA2^2^ and apoptotic spermatozoa (YO^+^) in teratoasthenozoospermic (case) and normozoospermic (control) men ((n=20/each

**Variables **	**Control group **	**Case group **	p-value*
Spermatozoa CMA3^+^	20.71±0.68	36.67±1.69	0.001
Spermatozoa HSPA2^+^	46.56±1.81	22.86±2.87	0.001
Intracellular O_2_^-^	16.35±2.7	33.69±1.43	0.001
Apoptotic spermatozoa	20.43±1.89	40.58±3.80	0.001

Values are mean±SE.

* independent-samples t-test

## Discussion

Infertility is a major reproductive health problem that affects about 15% of all reproduction age couples. Male factors are responsible for half of the cases (24). Despite the development of science in diagnostic methods, in some cases, the etiology of male infertility is still unknown. The present study conducted a comparison between the protamine deficiency, percentage of spermatozoa HSPA2^+^, and level of intracellular O_2_^−^ in patients suffering from terato-asthenozoospermic and normozoospermic men.

Our findings showed a statistically significant difference between case and control groups in terms of HSPA2. Case group experienced a significant decrease in the percentage of spermatozoa HSPA2^+^ in comparison to control group. The majority of other studies, such as studies conducted on infertile idiopathic oligo-teratozoospermia and patients with varicocele have shown a decrease in the level of HSPA2 (25, 26). 

Tian *et al* study indicated the correlation between the levels of HSPA2 expression in testis with spermatogenic impairment, and fertilization rate following intracytoplasmic injection treatment for azoospermic patients (20). Also, in a similar study, Moteiti *et al* have shown a decrease in the percentage of HSPA2 positive spermatozoa in infertile men by flow cytometry (16). Normally, residual cytoplasm is removed from spermatozoa during spermiation and failure in the release of excess residual cytoplasm (ERC) could cause cytoplasm retention (27). HSPA2 is involved in cytoplasmic extrusion during sperm maturation, and a decrease in the level of HSPA2 leads to cytoplasmic retention and abnormal spermatozoa morphology (15, 28). A positive correlation between ERC and ROS production has been shown by Gomez *et al* (17). 

It has also been reported that infertile men with varicocele have an increased level of excess residual cytoplasm and stimulated ROS production in semen (29). In fact, ERC activates the NADPH (Nicotinamide adenine dinucleotide phosphate) system through the hexose monophosphate shunt; this shunt is used by spermatozoa to supply electrons for ROS production, which further leads to oxidative stress (30). One of the main objectives of the present study was to investigate the intracellular O_2_^−^ in TA patients through flow cytometry. The results showed the higher levels of intracellular O_2_^−^ in the case group in comparison to control group. Multiple studies have shown similar results in both infertile patients (31) and experimental varicocele model (32). 

According to Pearson coefficient, there was a negative correlation between intracellular O_2_^−^ and sperm motility which could be due to the plasma membrane deficiency following oxidative stress. The excess in ROS levels can damage the sperm plasma membrane and its fluidity, thereby reducing membrane integrity and sperm motility (4, 33). According to the findings of the present study, decrease in HSPA2 and increase in intracellular O_2_^−^, might account as two characteristics of spermatozoa in TA patients. 

Apoptosis and abnormal chromatin condensation may have a negative effect on potential infertility. Therefore, we used CMA3 staining for detection of sperm protamine deficiency and yo - pro -1 was used to assess sperm apoptosis. The findings of this study demonstrated that TA patients have higher percentages of sperm apoptosis and spermatozoa with protamine deficiencies in comparison to control group. Multiple studies have shown the protamine deficiency in infertile or subfertile men (34, 35). The protamination process occurs during sperm nuclear compaction; protamine protects sperm DNA against the toxic effects of ROS, and protamine deficiencies may be considered as one of the main risk factors for sperm DNA fragmentation (36, 37). 

Also, DNA damage is a critical step in apoptosis; many studies have shown the relationship between DNA damage and abnormal sperm chromatin condensation (38, 39). According to our results, abnormal sperm chromatin condensation was another characteristic of spermatozoa from TA patients. On the other hand, HSPA2 plays a vital role in histon- protamine replacement; thus, one of the assumptions of the research was that the reduction of HSPA2 might cause chromatin abnormalities in TA samples (13). The results of a study showed a significant positive correlation between the percentage of CMA3 -positive sperm and HSPA2 in fertile men; however, the findings of the present research showed no correlation between these factors in two groups (16). Further studies with larger sample size covering different groups of infertile men are required to achieve more authentic, precise, and reliable findings. 

The findings of the present research showed increased rate of intracellular O_2_^−^ and decreased the percentage of sperm HSPA2^+^ in the samples of TA patients; however, there was no statistically significant correlation between them, which might be due to the other mechanisms for intracellular O_2_^−^ production in studied cases. 

## Conclusion

The findings of the present study showed that TA samples contain the lower percentage of HSPA2^–^ positive sperm, higher level of intracellular anion superoxide, and higher proportion of spermatozoa with abnormal chromatin packaging compare with normozoospermic men. Therefore, these factors might be considered as possible causes of infertility in these patients; it is worth mentioning that the assessment of these three factors is not only applicable to diagnostic design and treatment strategies in TA patients, it might also improve assisted reproductive success rates.

## Note

***This article extracted from the Ph.D. thesis. (Parvin Sabeti)***
